# Measuring educational leadership in Singapore: re-examining the psychometric properties of the Multifactor Leadership Questionnaire

**DOI:** 10.3389/fpsyg.2023.1280038

**Published:** 2023-11-17

**Authors:** John Chee Keng Wang, Woon Chia Liu, Ying Hwa Kee, Betsy Ng, Lilian Chua, Liang Hu

**Affiliations:** ^1^National Institute of Education, Nanyang Technological University, Singapore, Singapore; ^2^Zhejiang University, Hangzhou, China

**Keywords:** congeneric CFA, convergent validity, discriminant validity, MLQ-5X, transformational-transactional leadership

## Abstract

**Introduction:**

The Multifactor Leadership Questionnaire (MLQ-5X) has been used frequently to assess leadership in different settings. Despite its popularity, there are many critiques of the MLQ-5X such as its questionable multidimensional structure, lack of connection to the theory and the different factor structures of the measurement tool. The purpose of this study was to re-examine the psychometric properties of the MLQ-5X in the Singapore educational context using two datasets.

**Methods:**

A total of 872 teachers (40.1% male and 59.9% female) from 20 secondary schools in Singapore completed two sets of MLQ-5X, one set for their immediate reporting officer and one set for their school leaders.

**Results:**

Congeneric Confirmatory Factor Analysis, Rho’s coefficients, and AVE were used to analyze MLQ-5X’s convergent validity and internal consistency. After five items were deleted, the MLQ-5X showed acceptable internal consistency and convergent validity. Eight measurement models were tested with the original 36 items and the reduced items MLQ-5X. Latent factor correlation matrix with confidence intervals was used to assess the discriminant validity of the MLQ-5X. The results provided support for a nine first-order factors and three second-order factors model (transformation [IIA, IIB, IM, IS, IC, CR], transactional (MBEA), and non-leadership (MBEP and LF).

**Discussion:**

The discriminant validity of the hierarchical measurement model of MLQ-5X is supported using dataset 2.

## Introduction

Due to students’ consistent and high performance on international benchmarked student achievement tests like the Trends in Mathematics and Science Study (TIMSS), Progress International Reading Literacy Study (PIRLS), and Progress for International Student Assessment (PISA), Singapore’s educational system has recently gained recognition on a global scale. Many studies have been investigated contributing factors such as students’ motivation (e.g., Wang et al., 2019), teachers’ motivation (Wang et al., 2019) and classroom processes (e.g., Liu et al., 2020; 2023). One area that has been less studied is school leadership as a contributing factor to school processes and student achievement. It is a crucial field of research because it emphasizes the significance of the environments in which schools operate (Hallinger, 2018). Gopinathan and his colleagues (Gopinathan et al., 2008; Deng and Gopinathan, 2016) have attributed Singapore’s high performance in education system to its teacher quality, school leadership, system characteristics (such as standards, academic expectations, accountability measures) and education reform.

In educational leadership studies, most studies focused on school leaders or principals (Dimmock and Tan, 2013), the other key personnels such as heads of department or immediate supervisors are largely ignored. In the Singapore context, school leaders play important roles in supporting teachers in their professional learning and motivational outcomes (Lai et al., 2016; Lee and Nie, 2016). Thus, the perceptions of teachers toward their leaders could influence teachers’ behaviors in school and classroom. Lee and Nie (2016) found that both immediate supervisors and principals have an impact on teachers’ perceptions. It is thus valuable to examine leadership at both levels.

The Multifactor Leadership Questionnaire (MLQ-5X) has been used frequently to evaluate leadership in different settings (Avolio and Bass, 2004). A recent review of 29 studies by Bajcar and Babiak (2022) reported problematic psychometric properties including factor structures. They concluded that different studies used different factor structures, and despite the high correlations among some factors, in addition very few studies examined the validity of higher-order factors (Tepper and Percy, 1994; Densten and Sarros, 1997; Carless, 1998; Vandenberghe et al., 2002; Bajcar and Babiak, 2022). This has given rise to numerous criticisms of the MLQ-5X, including those about its dubious multidimensional structure, lack of relationship to the theory, and the measuring tool’s various factor structures (see Batista-Foguet et al., 2021). This indicates that the psychometric properties of the MLQ-5X still warrant attention. The purpose of this study was to re-examine the psychometric properties of the MLQ-5X in the Singapore educational context.

MLQ-5X is based on transformational leadership theory or full-range leadership (FRL). MLQ-5X was developed to assess leaders’ behavior, which includes transformational, transactional, and *laissez-faire* (Bass and Avolio, 1997; Avolio and Bass, 2004). Transformational leaders encourage followers to see beyond their own self-interest and go above and beyond (Antonakis et al., 2003). The transformational construct comprise of five factors: (1) idealized influence attributed (IIA) refers to conceptions of leaders as revered role models held by followers; (2) idealized influence behavior (IIB) refers to the observed actions of a leader who is believed to uphold high moral and ethical standards; (3) inspirational motivation (IM) is demonstrated in leaders when they encourage and inspire followers to show dedication to the group’s shared vision; (4) intellectual stimulation (IS) is when leaders encourage people to be innovative, challenge established practices, and suggest ways to make things better; and (5) individualized consideration (IC) refers to the capacity of a leader to assist, motivate, and direct subordinates (Bass, 1995; Avolio et al., 1999; Yukl, 2006).

Transactional leadership is when the leader bases his or her relationship with his or her followers on rewarding or disciplining them depending on their behavior and performance characteristics. There are three factors that define the transactional leader: (1) contingent rewards (CR) is how leaders reward followers based on results; (2) management by exception active (MBEA) is when the leaders uses negative reinforcement or corrective criticism on followers; and (3) management by exception passive (MBEP) exemplifies the traits of passive leaders who only act when expectations are not met (Bass, 1995; Avolio et al., 1999).

*Laissez-faire* (LF) leaders adopt a hands-off strategy and do not try to incentivize their followers or offer any other kind of internal or external reinforcement. Additionally, LF leaders avoid setting expectations, which causes them to put off making decisions and fixing problems (Northouse, 2012).

The MLQ-5X is suggested to be unpinned by nine first-order factors and three second-order factors (Bass and Avolio, 1997), as described above. The instrument has 45 items with 36 items measuring the nine factors as well as nine items measuring outcome of leadership variables such as effort, effectiveness, and satisfaction. Although many studies have supported the nine first-order factors measurement model of the MLQ-5X (e.g., Muenjohn and Armstrong, 2008; Xu et al., 2016), other studies have identified eight first-order factor model (Avolio et al., 1995), six first-order factors model (e.g., Vandenberghe et al., 2002; Bass et al., 2003), and five first-order factors model to be more suitable (Bycio et al., 1995). Some studies have identified certain items to have very low factor loadings and high error variances (e.g., Batista-Foguet et al., 2021; Moreno-Casado et al., 2021). This shows that there is a need to re-examine the MLQ-5X at the item-level before moving to the nine first-order factors structure. The current study uses a congeneric method of Confirmatory Factor Analysis (CFA) to investigate the constructs’ unidimensionality. Congeneric CFA refers to the method of evaluating several factors inside the framework of multifactor CFA models, or from single-factor CFA models to multifactor CFA models (Sinclair et al., 2006). This approach helps to determine the quality of the items and factors free from error disturbances from other factors. It is an appropriate method for item reduction.

Although MLQ-5X has been conceptualized as a hierarchical measurement model with three second-order factors (transformational, transactional, and lassie-faire), none of the previous studies have provided strong evidence for its existence. Only one study has validated the existence of three higher factors with six first-order factors model with the 36-items MLQ-5X (Avolio et al., 1999). Most studies have used the nine first-order factor model for MLQ-5X, but this is highly problematic as there are very high correlations between the factors measuring transformational leadership, the correlation coefficients were close to or higher than 0.90 (Xu et al., 2016; Batista-Foguet et al., 2021; Moreno-Casado et al., 2021). In a few studies, CR factor was merged with transformational leadership factors (Avolio et al., 1999; Vandenberghe et al., 2002; Alonso et al., 2010; Edwards et al., 2012) and MBEP was combined with LF to form one single factor (Bass et al., 2003; Heinitz et al., 2005; Kanste et al., 2007; Edwards et al., 2012). Hence, there is a need to examine the construct validity and discriminant validity of the MLQ-5X. In this study, we tested an alternative model with CR load to transformation leadership and MBEP and LF to form a non-leadership higher order factor (Model 8).

### Purposes of the study

The purpose of the present study was to examine the psychometric properties of MLQ-5X using two sets of data. Specifically, using a congeneric CFA technique, we intended to assess the unidimensionality of the four items associated with each MLQ component as well as their internal consistency and convergent validity. Secondly, we aimed to test the proposed measurement model (nine first-order factors with three higher-order factors) against seven alternative models, comparing the fit statistics from original 36 items models and the reduced items models. The discriminant validity will also be examined.

## Method

### Participants

A total of 872 teachers from 20 secondary schools in Singapore were recruited. There were 40.1% male teachers and 59.9% female teachers, these teachers were from different subjects ranging from languages to physical education. The teachers taught from less than 1 year to 44 years (mean number of years of teaching experience = 12.99 years). The teachers completed two sets of MLQ-5X, one set for their immediate reporting officer and one set for their school leaders. In this study, the responses related to immediate reporting officers will be used as dataset 1 and the responses to school leaders will be used as dataset 2.

### Procedures

Before beginning the investigation, the university’s Ethical Review Board’s ethical approval was sought and approved (IRB-2020-02-017). Permission to conduct research in school was granted by the Ministry of Education, Singapore. Following that, arrangements for survey administration were prepared and contacts with the school leaders of the schools were formed. Under the direction of a researcher, the questionnaires were distributed in a quiet classroom setting. Teachers were informed that their participation in the study was voluntary, that they could discontinue at any moment, and that the study would keep their answers confidential. The teachers provided informed consent and took approximately 30 min to complete the MLQ-5X.

### Measures

The MLQ-5X (Avolio et al., 1999) was used to capture both transformational leadership style ─ idealized influence attributes (IIA), idealized influence behavior (IIB), inspirational motivation (IM), intellectual stimulation (IS), individual consideration (IC); transactional leadership style ─ contingent reward (CR), management-by-exception (active) (MBEA), management-by-exception (passive) (MBEP); and *laissez-faire* (LF). There are 4 items each for the nine subscales. Responses were captured on a 5-point Likert scale (1 = Not at all to 5 = Frequently, if not always). There were also nine items measuring the outcomes of leadership but were not included in the analysis. The first part of the MLQ assessed the teachers’ perceptions of their immediate reporting officers’ leadership style (dataset 1) and the second part examined their perceptions toward their school principals (dataset 2).

### Data analysis

In the first dataset, the MLQ-5X’s convergent validity and internal consistency were estimated. As Cronbach’s (1951) coefficient alpha (α) presupposes that there are no measurement error covariances, this may be biased at the population level (Raykov, 1998). Rho’s coefficients were employed instead. Acceptable reliability is defined as a composite reliability coefficient (rho) of better than 0.60 (Bagozzi and Yi, 1988). We used the AVE index to check for convergent validity. The AVE index is a measure of shared or common variance in a latent variable. It is amount of variance that is captured by the latent variable in relation to the amount of variance due to measurement error (Dillion and Goldstein, 1984). The value needs to be greater than 0.50 to be accepted (Fornell and Larcker, 1981). Convergent validity examines the extent to which measures hypothesized to indicate the respective constructs load highly on the constructs (Bagozzi and Kimmel, 1995). Next, we conducted congeneric CFA on each of factor of MLQ-5X using EQS for Windows 6.4 (Bentler, 2006). Following reduction of items, a second congeneric CFA was conducted in the affected factors.

Various criteria were used, to evaluate a good model fit. They were: Satorra-Bentler scaled Chi-square statistics, robust non-norm fit index (NNFI), robust root mean square error of approximation (RMSEA), robust comparative fit index (CFI), and robust IFI. These robust indices and scaled chi-square outperform the ML indices when the data are non-normal (Curran et al., 1996). Yu and Muthen (2002) recommend that a good fit is achieved when the robust RMSEA is 0.05 or less, and when robust fit indices are close to or greater than 0.95.

Next, CFA was carried out on the MLQ-5X to investigate its factorial validity. Eight measurement models were compared. The first seven models were selected based on the review by Bajcar and Babiak (2022) as the most common measurement models being tested. The first model (Model 1) was a nine first-order factors model (IIA, IIB, IM, IS, IC, CR, MBEA, MBEP, LF). The second model (Model 2) was a six first-order factors model [(IIA, IIB, IM), IS, IC, CR, MBEA, (MBEP, LF)]. The third model (Model 3) was a six first-order factors model (as with Model 2) with two high order factors [transformation (IIA, IIB, IM), IS, IC, CR], versus transactional [MBEA, (MBEP, LF)]. The fourth model (Model 4) was a six first-order factors (as with Model 2) with three higher order factors model {transformation [(IIA, IIB, IM), IS], transactional (IC, CR), and non-leadership MBEA, (MBEP, LF)}. The fifth model (Model 5) was a hierarchical model comprising nine first-order factors and two higher-order factors [transformational (IIA, IIB, IM, IS, IC) versus transactional (CR, MBEA, MBEP, LF)]. The sixth model (Model 6) was a hierarchical model with the nine first-order factors and three higher-order factors [transformation (IIA, IIB, IM, IS, IC), transactional (CR, MBEA, MBEP), and *Laissez-faire* (LF)]. This is the hierarchical structure proposed by Avolio et al. (1995). Model 7 was a hierarchical model with the nine first-order factors and four higher-order factors (Model 7, transformation ([IIA, IIB, IM, IS, IC], CR, MBEA, non-leadership [MBEP, LF])). Finally, Model 8 was a hierarchical model with the nine first-order factors and three higher-order factors [transformation (IIA, IIB, IM, IS, IC, CR)], transactional (MBEA), and non-leadership (MBEP and LF). The CFA on the eight models was conducted twice. One with the original 36 items and one after item reduction for comparisons.

The confidence intervals of the latent factor correlation between each pair of components were analyzed to test for discriminant validity (*ϕ*-coefficients). When the correlations are significantly below unity (1.00), the measure’s discriminant validity is supported (Bagozzi, 1981).

To validate the modified measurement model of the MLQ-5X, we used a second dataset and conducted CFAs on those models that obtain satisfactory model fits.

## Results

The internal consistency coefficients (rho), AVE and the fit statistics and factor loadings of the congeneric CFA are shown in Table 1. While all the subscales had satisfactory rho’s coefficients of 0.70 and above, four of the nine subscales of MLQ-5X showed unsatisfactory AVE values (<0.50). The factor loadings of some of the items are lower than 0.60, the low factor loadings indicate low shared variance with the constructs measured. The NNFI and chi-square also indicate some misspecifications of the items within the factors (IB, IS, IC, CR and MBEP). After initial consideration, five items (IIA4, IC3, CR2, BMEA2, BMEP3) were deleted, and the internal consistency coefficients and congeneric CFA was repeated. As shown in Table 2, the AVE of the five factors improved and the fit statistics and chi-square are almost perfect. This provides support for the reduction of the five items. The convergent validity and internal consistency of the MLQ-5X are supported.

**Table 1:** Reliability coefficients, fit indices and item loadings of each single factor.

Model (scale/item coding)	Loadings	Rho	AVE	*χ*^2^(2)	NNFI	CFI	IFI	SRMR	RMSEA (90% CI)
Idealized attributes	0.75; 0.79; 0.91; 0.55	0.85	0.58	3.66	0.996	0.999	0.999	0.011	0.033 (0.000, 0.087)
Idealized behaviors	0.64; 0.79; 0.71; 0.75	0.81	0.53	20.14	0.945	0.982	0.982	0.025	0.110 (0.070, 0.156)
Inspirational motivation	0.73; 0.78; 0.78; 0.70	0.84	0.56	9.74	0.980	0.993	0.993	0.016	0.071 (0.031, 0.119)
Intellectual stimulation	0.68; 0.64; 0.82; 0.77	0.82	0.52	28.77	0.922	0.974	0.974	0.032	0.134 (0.093. 0.179)
Individual consideration	0.78; 0.61; 0.56; 0.86	0.80	0.51	26.59	0.924	0.975	0.975	0.036	0.128 (0.087, 0.173)
Contingent reward	0.63; 0.53; 0.67; 0.68	0.73	0.40	11.77	0.948	0.983	0.983	0.024	0.081 (0.041, 0.128)
Management-by-exception (active)	0.62; 0.45; 0.75; 0.61	0.70	0.38	4.58	0.985	0.995	0.985	0.017	0.041 (0.000, 0.093)
Management-by-exception (passive)	0.66; 0.77; 0.48; 0.78	0.77	0.47	39.76	0.862	0.954	0.954	0.042	0.159 (0.118, 0.203)
*Laissez-faire*	0.69; 0.77; 0.67; 0.62	0.78	0.48	0.09	1.00	1.00	1.00	0.002	0.000 (0.000, 0.000)

NNFI, Non-Normed Fit Index; CFI, Comparative Fit index; GFI, Goodness-of-Fit Index.

RMSEA, Root Mean Square Error of Approximation; CI, confidence interval for relevant point estimates.

**Table 2:** Reliability coefficients, fit indices and item loadings of the single factor with item deletion.

Model (scale/item coding)	Loadings	Rho	AVE	χ^2^(2)	NNFI	CFI	IFI	RMSEA (90% CI)
Idealized attributes	0.74; 0.78; 0.91	0.86	0.67	0.02	1.003	1.000	1.001	0.000 (0.000, 0.000)
Individual consideration	0.78; 0.60; 0.86	0.80	0.57	0.97	1.002	1.000	1.001	0.000 (0.000, 0.058)
Contingent reward	0.67; 0.63; 0.69	0.70	0.44	0.01	1.007	1.000	1.003	0.000 (0.000, 0.000)
Management-by-exception (active)	0.62; 0.76; 0.61	0.70	0.44	0.37	1.007	1.000	1.003	0.000 (0.000, 0.042)
Management-by-exception (passive)	0.69; 0.82; 0.72	0.78	0.55	0.00	1.003	1.000	1.001	0.000 (0.000, 0.000)

NNFI, Non-Normed Fit Index; CFI, Comparative Fit index; GFI, Goodness-of-Fit Index.

RMSEA, Root Mean Square Error of Approximation; CI, confidence interval for relevant point estimates.

Table 3 shows the fit indices of the eight measurement models with full MLQ-5X items and Table 4 shows the fit statistics of the eight models after five items were deleted. When the modification indices of all the measurement models were examined, it was found that two error variances correlated with each other strongly (IM4 and CR4), the two error variances are allowed to be correlated in the estimation. None of the eight measurement models fit the data before item deletion (see Table 3). Table 4 shows Models 1, 7 and 8 after 5 items are deleted have satisfactory fit indices.

**Table 3:** Results of the CFAs across models with no item deletion.

Model	Scaled *χ*^2^	df	NNFI	CFI	IFI	RMSEA (CI)
Model 1: 9 factors	1539.45	558	0.879	0.893	0.893	0.051 (0.048, 0.054)
Model 2: 6 factors	1754.92	579	0.860	0.871	0.872	0.055 (0.052, 0.058)
Model 3: 6 first-order factors and 2 higher order factors	1798.36	587	0.858	0.867	0.868	0.055 (0.053, 0.058)
Model 4: 6 first-order factors and 3 higher order factors	1789.93	585	0.858	0.868	0.869	0.055(0.052, 0.058)
Model 5: 9 first-order factors and 2 higher order factors	1764.28	584	0.861	0.871	0.871	0.055 (0.025, 0.058)
Model 6: 9 first-order factors and 3 higher order factors	1630.89	582	0.876	0.885	0.8786	0.052 (0.049, 0.055)
Model 7: 9 first-order factors and 4 higher order factors	1616.37	579	0.876	0.886	0.887	0.052 (0.049, 0.055)
Model 8: 9 first-order factors and 3 higher order factors (CR to transformational)	1750.18	582	0.862	0.872	0.873	0.055 (0.052, 0.058)

NNFI, Non-Normed Fit Index; CFI, Comparative Fit index; GFI, Goodness-of-Fit Index; RMSEA, Root Mean Square Error of Approximation; CI, confidence interval for relevant point estimates.

**Table 4:** Results of the CFAs across models after item deletion.

Model	Scaled *χ*^2^	df	NNFI	CFI	IFI	RMSEA (CI)
Model 1: 9 factors	852.97	397	0.934	0.944	0.944	0.041 (0.037, 0.045)
Model 2: 6 factors	1110.82	418	0.905	0.915	0.915	0.050 (0.046, 0.053)
Model 3: 6 first-order factors and 2 higher order factors	1138.35	426	0.904	0.912	0.913	0.050 (0.046, 0.053)
Model 4: 6 first-order factors and 3 higher order factors	1126.51	424	0.905	0.914	0.914	0.050(0.046, 0.083)
Model 5: 9 first-order factors and 2 higher order factors	1070.16	423	0.912	0.920	0.921	0.048 (0.044, 0.051)
Model 6: 9 first-order factors and 3 higher order factors	1786.51	423	0.815	0.832	0.833	0.069 (0.066, 0.072)
Model 7: 9 first-order factors and 4 higher order factors	1025.96	418	0.917	0.925	0.926	0.046 (0.043, 0.050)
Model 8: 9 first-order factors and 3 higher order factors (CR to transformational)	924.98	418	0.931	0.938	0.938	0.042 (0.039, 0.046)

NNFI, Non-Normed Fit Index; CFI, Comparative Fit index; GFI, Goodness-of-Fit Index.

RMSEA, Root Mean Square Error of Approximation; CI, confidence interval for relevant point estimates.

Next, the discriminant validity of the MLQ-5X is assessed using the latent factor correlation matrix with confidence intervals (see Table 5). It was found that the confidence intervals (CI) of the latent factor correlations between IIB and IM, IC and IIA, CR and IC, and MBEP and LF exceeded 1.00. This shows that IIA, IIB, IC, IM, and CR are not empirically justified as independent constructs. Therefore, Models 1 and Model 7 are not suitable measurement models for the MLQ-5X due to the lack of discriminant validity of the first-order constructs.

**Table 5:** Latent factor correlations with confidence intervals (sample 1).

Variable	1	2	3	4	5	6	7	8
1. Idealized attributes	1.00							
2. Idealized behaviors	. 86^*^ (0.02) 0.82, 0.90	1.00						
3. Inspirational motivation	. 88^*^ (0.02) 0.84, 0.92	. 99^*^ (0.01) 0.97, 1.01	1.00					
4. Intellectual stimulation	. 87 (0.02) 0.83, 0.91	. 92^*^ (0.02) 0.88, 0.96	. 91^*^ (0.02) 0.87, 0.95	1.00				
5. Individual consideration	. 97^*^ (0.02) 0.93, 0.1.01	. 84^*^ (0.02) 0.80, 0.88	0.90^*^ (0.02) 0.86, 0.94	0.93^*^ (0.02) 0.89, 0.97	1.00			
6. Contingent reward	. 94^*^ (0.02) 0.90, 0.98	. 93^*^ (0.03) 0.87, 0.99	0.92^*^ (0.02) 0.88, 0.96	0.93^*^ (0.03) 0.87, 0.99	1.00^*^ (0.02) 0.96, 1.04	1.00		
7. Management-by-exception (active)	−0.38^*^ (0.04) −0.46, −0.30	−0.10^*^ (0.05) −0.20, 0.00	. -0.22^*^ (0.05) −0.32, −0.12	−0.24^*^ (0.05) −0.34, −0.14	−0.33^*^ (0.05) −0.43, −0.23	−0.26^*^ (0.05) −0.36, −0.16	1.00	
8. Management-by-exception (passive)	−0.71^*^ (0.03) −0.77, −0.65	−0.69^*^ (0.04) −0.77, −0.61	−0.69^*^ (0.04) −0.77, −0.61	−0.68^*^ (0.04) −0.76, −0.60	−0.70^*^ (0.04) −0.78, −0.62	−0.72^*^ (0.04) −0.80, −0.64	0.40^*^ (0.05) 0.30, 0.50	1.00
9. *Laissez-faire*	−0.78^*^ (0.03) −0.84, −0.72	−0.71^*^ (0.03) −0.77, −0.65	−0.69^*^ (0.04) −0.77, −0.61	−0.68^*^ (0.04) −0.76, −0.60	−0.73^*^ (0.03) −0.79, −0.67	−0.72^*^ (0.04) −0.80, −0.64	0.37^*^ (0.05) 0.36, 38	0.97^*^ (0.02) 0.93, 1.01

^*^p < 0.05. In each cell, first row = latent factor correlation, second row = SE of latent correlation coefficient, last row = correlation confidence intervals within plus/minus 2 SE.

**Figure 1:**
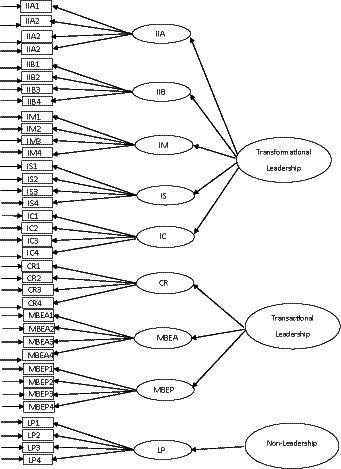
Original proposed factorial structure of the MLQ-5X (36-items).

**Figure 2:**
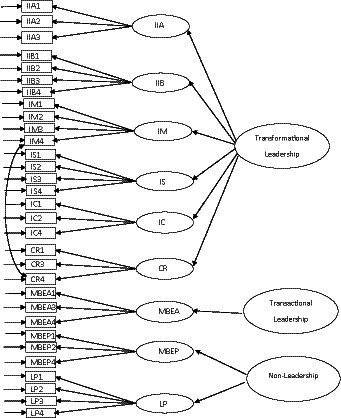
Finalized factorial structure of the MLQ-5X (31-items).

The next step of the analysis was to use dataset 2 to check the factorial structure of Model 8. The fit indices were adequate (Scaled *χ*^2^ = 976.68, df = 417; NNFI = 0.932; CFI = 0.939; IFI = 0.939; RMSEA = 0.042, CI of RMSEA = 0.038 and 0.045). Therefore, we concluded that the MLQ-5X is better represented with a nine first-order factors and three second-order factors transformation (IIA, IIB, IM, IS, IC and CR), transactional (MBEA), and non-leadership (MBEP and LF). The latent factor correlations with confidence intervals among the three higher order factors are presented in Table 6. Figure 1 shows the original proposed factor structure of the MLQ-5X (Avolio and Bass, 2004) and Figure 2 shows the final factor structure of the revised MLQ-5X with 31 items. As all the latent factor correlation coefficients are significantly lower than 1.00, the discriminant validity of the hierarchical measurement model of MLQ-5X is supported.

**Table 6:** Latent factor correlations with confidence intervals of the higher order factors (sample 2).

	1	2
Transformational		
Transactional	−0.47^*^ (0.04) −0.55, −0.39	
Non-leadership	−0.75^*^ (0.04) −0.83, −0.67	0.64^*^ (0.04) 0.56, 0.72

^*^p < 0.05. In each cell, first row = latent factor correlation, second row = SE of latent correlation coefficient, last row = correlation confidence intervals within plus/minus 2 SE.

## Discussion

Leadership in schools has contributed much to the effectiveness of Singapore education success. However, there is a lack of research in this domain, particularly at the middle management level. The present study aimed to assess the psychometric properties of an established yet problematic leadership questionnaire, MLQ-5X using two sets of data. The teachers responded to the MLQ-5X, one for their immediate supervisors, and one for their school principals.

We tested the internal consistency, convergent validity, discriminant validity and tested several measurement models. Previous studies commonly used the Cronbach’s (1951) coefficients to examine reliability and mean scores of the subscale to compute correlations among the factors. These methods are problematic as it assumes that there is no measurement error covariances between the items in a factor. The use of rho’s coefficient and latent factors are more appropriate (Raykov, 1998).

The results supported the internal reliability of the nine factors of MLQ-5X. However, four out of nine factors had unsatisfactory AVE values of lower than 0.50. If a construct exhibits convergent validity, according to Fornell and Larcker (1981), the average variance extracted (AVE) must be at least.50 (this means the variance explained by the construct is more than measurement error). Two items also had low factor loadings from the congeneric CFA. After the deletion of five items (IIA4, IC3, CR2, BMEA2, BMEP3), the results of the congeneric CFA supported the unidimensionality of the five factors (IIA, IC, CR, BMEA, BMEP). Recently, Batista-Foguet et al. (2021) have conducted a qualitative content analysis of the wording of the four items linked to each of the nine MLQ factor. They suggested that some of the items are problematic within CR, MBEA, MBEP. For example, three of the items in CR factor includes economic and emotional exchange, but one item is linked to an individual’s commitment to pursue performance standards. Together with the results of the congeneric CFA, the reduction of the items is justifiable.

This study provided a clear method of item reduction through congeneric CFA, which is a novel approach (Markland and Ingledew, 1997). Congeneric CFA emphasizes a methodological improvement to the MLQ-5X whilst considering the integrity of the construct. In this study, one item was deleted from each of the five factors of MLQ-5X and the reduced factor structure exhibited better fit by keeping items that are truly working well in the model.

We compared eight measurement models of the MLQ-5X in this study. With the original MLQ-5X, none of the eight measurement models provided adequate fit. After the five items were deleted, the results showed that the nine first-order factors model (Model 1), nine first-order factors and two higher order factors (Model 7) and nine first-order factors and three higher order factors (Model 8) may be suitable according to the fit indices. However, Models 1 and 7 lack discriminant validity in that some of the first-order factors are not empirically independent constructs. Thus Model 8 is the most valid measurement model.

The use of a second dataset supported Model 8, a nine first-order factors and three higher-order factors model. In this model, CR is grouped as transformational leadership factor, MBEA as transactional factor, and MBEP and LF as non-leadership factor. The factor structure and discriminant validity are supported. MBEP and LF have been grouped as non-leadership factor in many previous studies using CFA (e.g., Rowold, 2005; Moreno-Casado et al., 2021), so it is not an issue. Should CR be grouped under transformational leadership? The content analysis of Batista-Foguet et al. (2021) shows that the wording of the items in CR should be grouped as transformational leadership factor, rather than transactional leadership. The argument for this can be explained from Vroom’s (1964) Expectancy Theory of Motivation. People can be motivated if they achieved the outcomes in the workplace and receiving rewards is a way to prove that they have achieved the outcomes that they valued. Another theory that can explain how contingent reward is linked to intrinsic motivation is cognitive evaluation theory (Ryan and Deci, 2017). If one perceives that the rewards enhance his/her sense of competency and autonomy (informational functional significance), his/her intrinsic motivation for the task will be enhanced. It is thus not surprising that CR can be grouped into transformational leadership. Previous studies have also supported the inclusion of CR with the five transformation leadership dimensions (Lowe et al., 1996; Vandenberghe et al., 2002; Judge and Piccolo, 2004; Boamah and Tremblay, 2019). One other finding is that two of the items in MLQ-5X seem to be related (IM4 and CR4). A careful analysis of the two items shows similarity, both items relate to confidence and satisfaction when outcomes are achieved.

In conclusion, the current study shows evidence of a nine first-order factors and three higher-order factors measurement model for the MLQ-5X. The findings affirm that the MLQ-5X is an appropriate measurement tool to assess leadership in the educational setting. With a proper measurement tool in place, researchers can then move on to look at leadership in three dimensions (transformational, transactional, and non-leadership). For example, the impact of different types of leaderships on school climate, teachers’, and students’ outcomes.

There are several possible limitations that need to be acknowledged. First, some researchers may question the use of CFA in item deletion of measurement tool. However, according to Larwin and Harvey (2012), a congeneric CFA is appropriate for item deletion with established questionnaires, while exploratory factor analysis (EFA) is more pertinent for examining new questionnaires. In view of this, congeneric CFA is thought to be more suited than EFA since the MLQ-5X is regarded as a well-established inventory to measure characteristics of leadership. Second, this study did not examine the concurrent validity and predictive validity of the refined MLQ-5X. Future studies should investigate the MLQ-5X refinement’s concurrent and predictive validity in relation to other factors such school atmosphere, teacher motivation, performance, and other outcome variables. Thirdly, this study did not examine the invariance measurement structure of the MLQ-5X between gender and years of teaching experience. Future studies should examine the invariance of the new measurement model. Fourthly, the MLQ-5X was administered to teachers belonging to different departments and different schools, with each teacher completing the inventory to describe their immediate supervisors and principals. The nature of the data is hierarchical and thus the multilevel effects of the MLQ-5X need to be examined in future studies. Finally, the longitudinal score stability at the level of the latent construct could be examined by testing the longitudinal factor invariance (Conroy et al., 2003).

## Data availability statement

The raw data supporting the conclusions of this article will be made available by the authors, without undue reservation.

## Ethics statement

The studies involving humans were approved by Nanyang Technological University IRB. The studies were conducted in accordance with the local legislation and institutional requirements. The participants provided their written informed consent to participate in this study.

## Author contributions

JW: Conceptualization, Investigation, Methodology, Writing – original draft. WL: Funding acquisition, Investigation, Writing – review & editing. YK: Writing – review & editing. BN: Writing – review & editing. LC: Writing – review & editing. LH: Writing – review & editing.
